# Tumor Microenvironment of Esophageal Cancer

**DOI:** 10.3390/cancers13184678

**Published:** 2021-09-18

**Authors:** Lars M. Schiffmann, Patrick S. Plum, Hans F. Fuchs, Benjamin Babic, Christiane J. Bruns, Thomas Schmidt

**Affiliations:** Department of General, Visceral, Cancer and Transplantation Surgery, Faculty of Medicine with University Hospital Cologne, University of Cologne, 50931 Cologne, Germany; lars.schiffmann@uk-koeln.de (L.M.S.); patrick.plum@uk-koeln.de (P.S.P.); Hans.fuchs@uk-koeln.de (H.F.F.); benjamin.babic@uk-koeln.de (B.B.); christiane.bruns@uk-koeln.de (C.J.B.)

**Keywords:** esophageal cancer, esophageal adenocarcinoma, esophageal squamous cell cancer, tumor microenvironment, cancer-associated fibroblasts, tumor angiogenesis, tumor-associated macrophages, immunotherapy

## Abstract

**Simple Summary:**

Esophageal cancer is one of the top ten most deadly cancers. Even when diagnosed in a curable stage, patients prognosis poor. One of the parameters that is very relevant for long-term survival is response to radio(chemo)therapy prior surgery. Complete response rates are between 24 and 50 percent. This puts more than a half of every esophageal cancer patient that is diagnosed in a non-metastasized stage at high risk of recurrence. To improve response rates of treatment regimens prior curative surgery is, therefore, a major challenge in treating esophageal cancer. Not only the response of the cancer cell itself to cancer therapy is determining patients’ fate. Cells around the tumor cells called the tumor microenvironment that together with the cancer cell constitute a malignant tumor are also involved in tumor progression and therapy response. This review depicts the most important parts of the esophageal cancer microenvironment, evaluates chances and challenges of current already established therapeutic concepts that target this microenvironment. It furthermore elucidates specific pathways that are potential valuable targets in the future.

**Abstract:**

Esophageal cancer is among the top ten most deadly cancers worldwide with adenocarcinomas of the esophagus showing increasing incidences over the last years. The prognosis is determined by tumor stage at diagnosis and in locally advanced stages by response to (radio-)chemotherapy followed by radical surgery. Less than a third of patients with esophageal adenocarcinomas completely respond to neoadjuvant therapies which urgently asks for further strategies to improve these rates. Aiming at the tumor microenvironment with novel targeted therapies can be one strategy to achieve this goal. This review connects experimental, translational, and clinical findings on each component of the esophageal cancer tumor microenvironment involving tumor angiogenesis, tumor-infiltrating immune cells, such as macrophages, T-cells, myeloid-derived suppressor cells, and cancer-associated fibroblasts. The review evaluates the current state of already approved concepts and depicts novel potentially targetable pathways related to esophageal cancer tumor microenvironment.

## 1. Introduction

Esophageal cancer is among the top ten most common and most deadly cancers worldwide [1,2]. Concerning the western world, the incidence of esophageal squamous cell cancer (ESCC) is decreasing while the occurrence of esophageal adenocarcinomas including adenocarcinomas of the esophagogastric junction (EAC) is rapidly increasing, which overall significantly accelerated the incidence of the disease. Mostly detected at locally advanced or already metastasized stages the prognosis is still detrimental in many cases though great advances in (multimodal) treatment strategies have been achieved over the last decades. Detected in a non-metastasized stage, the multimodal treatment consists of perioperative chemotherapy or neoadjuvant radiation and a radical oncologic surgical therapy, except for early stage pT1 cases (which qualify for either endoscopic resection or primary surgery). The overall survival in large randomized controlled trials examining the value of neoadjuvant radiation before surgery or perioperative chemotherapy improved to 24–50 months in the groups of multimodally treated patients, which lead to the implementation of these treatment strategies in international and national guidelines [3,4]. Pathological complete response rates to chemoradiotherapy were 23% for EAC patients and 49% for ESCC patients in the CROSS trial and 16% in the FLOT4 trial in adenocarcinomas of the esophagogastric junction [3,5]. Results from these studies showed that response to neoadjuvant treatment is highly relevant for overall survival with complete responders showing a 5-year survival rate of over 70% in contrast to poor responders with at least 50% residual tumor in the specimen having a 5-year survival rate of around 20% [4]. Patients with adenocarcinoma of the esophagogastric junction were found to have a significantly longer disease-free survival after pathological complete response after preoperative chemotherapy [6].

This shows that one major challenge in the treatment of esophageal cancer at least for locally advanced stages that are treated with curative intent is the improvement of response rates. Although one strategy was the implementation of more intense conventional chemotherapeutic regimens as in the FLOT protocol [7,8], another is to develop new targeted therapies that complement conventional therapy [9,10]. To do so a deep mechanistic understanding of each specific tumor entity is essential.

A malignant tumor is composed not only of tumor cells but to a large part of adjacent non-malignant cells, the so-called tumor stroma. The interaction between tumor cells and stromal cells, between stromal cells among themselves and between cells from different areas of tumor tissues is described as the tumor microenvironment [11]. This article aims to give an overview of the tumor microenvironment in esophageal cancer with a specific focus on the mechanistic backgrounds that give the rationale for tumor microenvironment targeted therapies.

## 2. Endothelial Cells and Tumor Angiogenesis

When a malignant lesion reaches a critical size, diffusion does not suffice to reach the center of the tumor with oxygen and nutrients. The tumor core becomes hypoxic which leads to the stabilization of hypoxia-inducible factor-1α (HIF-1α), a transcription factor that then further induces the upregulation of several pro-angiogenic genes encoding for cytokines and growth factors that mediate an angiogenic response. This process has been described as the angiogenic switch. Until now vascular endothelial growth factor (VEGF) is considered to be the most potent pro-angiogenic factor. VEGF-receptors, mainly VEGFR2, with their downstream signaling pathways mediate proliferation, migration, and cell survival in endothelial cells under physiologic and pathologic conditions [12]. Many drugs that target pro-angiogenic signaling cues have been developed in the last 2 decades with bevacizumab (bev) a monoclonal antibody against VEGF being the most prominent one. Bevacizumab prolonged progression-free survival and overall survival in patients with metastatic colorectal cancer in combination with conventional chemotherapy compared to chemotherapy alone [13]. Bevacizumab became standard of care in many tumor entities mostly in a palliative setting, but also in neoadjuvant treatment strategies, e.g., for the multimodal treatment of ovarian cancer.

Experimentally in mice, anti-angiogenic therapies can impressively reduce tumor vascularization, thereby ‘starving’ tumors to death, following Judah Folkman’s hypothesis, which stimulated the field of angiogenesis research in the 1970s.

On a tumor histological and cellular level experimental work [14,15,16,17] could demonstrate the mechanisms underlying the efficacy of anti-VEGF therapies is more complex than initially believed. Anti-angiogenic therapy in patients does indeed reduce the number of tumor vessels [18] but also reduces the tumor interstitial fluid pressure making the tumor more sensitive for drug uptake. The beneficial effect on tumor interstitial fluid is believed to result from improved blood vessel structure and function due to VEGF withdrawal. This phenomenon was summarized by Rakesh Jain as the vascular normalization theory [19].

In esophageal cancer, VEGF seems to play a pivotal role in tumor progression similar to other entities. Shimada et al. examined VEGF serum levels of patients with esophageal squamous cell cancer (ESCC) and found that serum levels were correlated with increased tumor stage and prognosis [20]. Interestingly, high serum VEGF levels predicted poor response to radiochemotherapy. These data suggest a strong rationale to pharmacologically deplete serum VEGF levels in these patients; further translational and clinical research should be done to evaluate anti-angiogenic treatment in combination with conventional radiochemotherapy in this particular tumor entity. Surprisingly not a single clinical trial involving anti-angiogenic treatment was conducted in ESCC following these promising initial translational data. However, in esophageal adenocarcinoma and carcinoma of the esophagogastric junction intense effort has been done to evaluate anti-angiogenic treatments [21].

First, bevacizumab was tested in an international multi-center randomized trial (AVAGAST). This study compared capecitabine-cisplatin chemotherapy in conjunction with bevacizumab as first-line treatment in patients with advanced gastric cancer [22]. Though the study did not reach its primary endpoint, which was an improvement in overall survival, the addition of bevacizumab to capecitabine-cisplatin significantly improved median progression-free survival (6.7 months vs. 5.3 months; hazard ratio, 0.80; *p* < 0.0037) and overall response rate (46.0% vs. 37.4%; *p* = 0.0315). Overall, the AVAGAST trial underscored the role of VEGF signaling and its therapeutic potential in advanced gastric cancer and carcinomas of the esophagogastric junction, the latter representing 45 from 200 analyzed patients in Europe [22]. Preplanned subgroup analysis within the AVAGAST trial suggested that Asian patients do not benefit from the addition of bevacizumab to conventional chemotherapy. This was confirmed by the AVATAR study which showed no effect of bevacizumab when added to capecitabine-cisplatin therapy compared to capecitabine-cisplatin therapy plus placebo in Chinese patients [23]. Accordingly, there are potential ethical differences in the sensitivity towards VEGF-blockade which might be a result of differences concerning tumor immunological gene expression signature [24]. It is further noteworthy that both, in the AVAGAST trial and the AVATAR study the majority of included patients suffered from gastric cancer with significantly fewer patients with carcinomas of the esophagogastric junction being randomized. This is a potential bias in both studies which probably leads to the underestimation of beneficial effects of VEGF inhibition in esophagogastric junction tumors.

Accordingly, it was not surprising that bevacizumab failed to improve survival outcomes in patients with resectable esophagogastric adenocarcinoma patients that were randomized to receive peri-operative epirubicin, cisplatin, and capecitabine chemotherapy or chemotherapy plus bevacizumab prior to surgery [25]. The patients included in this study consisted of around a third of patients with gastric cancer supporting the hypothesis that tumor location has potential relevance for the efficacy of VEGF inhibition in gastro-esophageal adenocarcinoma. These results claim for further examination as subgroup analysis concerning tumor location was inconsistent in between different types of Siewert tumors with GEJ type III tumors showing a beneficial outcome after chemotherapy plus bevacizumab and GEJ Type I and II tumors showing no difference [25].

In parallel ramucirumab, a fully humanized monoclonal antibody that counteracts VEGF binding do VEGF-R2, entered clinical trials. The REGARD study investigating the effect of ramucirumab as a monotherapy compared to placebo in previously treated patients with advanced gastric cancer or adenocarcinomas of the esophagogastric junction that showed progressive disease after first-line treatment. Ramucirumab significantly prolonged overall survival in these patients and, therefore, is the first and until now only anti-angiogenic agent that prolonged survival administered as a single agent without additional chemotherapy [26]. Additionally, ramucirumab was evaluated in a large multi-center randomized phase 3 trial as second-line treatment in combination with paclitaxel [27]. Results from the RAINBOW study demonstrated an improvement in overall survival in patients treated with ramucirumab plus paclitaxel compared to placebo plus paclitaxel (median 9.6 months vs. 7.4 months; hazard ratio, 0.807; *p* = 0.017). These results together lead to the implementation of ramucirumab in national and international guidelines for the second-line treatment of advanced gastric and esophagogastric junctional adenocarcinomas [28].

One of the major unanswered questions or challenges in the field of tumor-related angiogenesis research is, however, to identify mechanisms of response or resistance and to develop suitable biomarkers based on these findings.

Tremendous efforts have been spent on uncovering mechanisms of escape and resistance to anti-angiogenic treatments involving cellular, non-cellular, stromal, and tumor cell inert mechanisms in other tumor entities than esophageal cancer [18,29,30,31,32,33,34,35]. To our knowledge, no experimental work has been done specifically on esophageal or esophagogastric cancer which might simply be related to the fact that there is a lack of suitable animal models for this disease, especially for esophageal cancers. On a translational basis, serum samples from the AVAGAST trial were evaluated for the potential role of angiopoetin-2 as a predictive biomarker for bevacizumab-containing therapies [36]. This hypothesis was generated based on positive findings in colorectal cancer patients [37]. Other than in colorectal cancer patients, angiopoietin-2 had no value in response to bevacizumab concerning survival. However, high serum angiopoietin-2 levels are associated with a poor survival outcome in patients randomized for the AVAGAST trial which again emphasizes the role of angiogenesis in esophagogastric junction tumors [36,38]. Further research on other angiogenesis mediating signaling pathways is needed to develop alternative or complementary approaches to pharmacologically target tumor angiogenesis in esophageal cancer [39].

**Figure 1:**
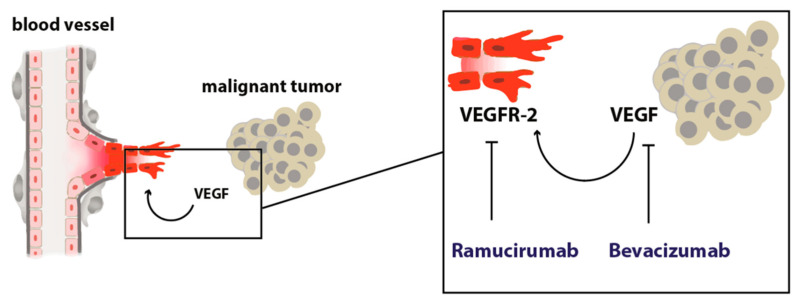
Scheme depicting the angiogenic switch. A malignant tumor secretes pro-angiogenic growth factors that stimulate angiogenesis by acting on endothelial cells with pro-migratory, proliferative, and anti-apoptotic, pro-survival signals.

In summary, inhibition of tumor angiogenesis with ramucirumab in (distal) esophageal cancer is a valuable option in a palliative setting in combination with chemotherapy or is administered as a single agent. The fact that ramucirumab is potently prolonging overall survival, which bevacizumab was not able to achieve in clinical trials, points towards a very interesting connection that warrants further investigation. Bevacizumab eliminates serum and tissue VEGF by binding the growth factor itself. In theory, this does not only inhibit VEGF effects that are mediated via VEGF-R2 on endothelial cells (Figure 1) but also inhibits other signals that are mediated by other VEGF receptors, such as VEGF-R1 on endothelial cells, or other cells of the tumor microenvironment like tumor-infiltrating immune cells like macrophages or monocytes. For example, VEGF-R1 on macrophages was recently found as a crucial player in metastasis and progression. A high percentage of VEGF-R1 positive immune cells within colorectal metastasis predicts worse outcomes in patients [35]. It is known from endothelial cell sprout differentiation that VEGF-R2 and VEGF-R1 are somehow reciprocally regulated meaning that VEGF-R1 signaling can limit VEGF-R2 signaling by acting as a decoy receptor. Eliminating VEGF from the system could interact with these processes in ways that are context dependently not always beneficial or complex to predict in every oncological situation. These thoughts are certainly hypothetical but might explain the differences in the efficacy of bevacizumab and ramucirumab.

Another open question is whether other promising novel anti-angiogenic approaches that have been explored preclinically over the past decades are potent and tolerable enough to enter the clinical application. Especially, concepts that target endothelial metabolism produce promising experimental results but have to be further evaluated in early phase clinical trials [40,41,42,43,44,45].

## 3. Cancer-Associated Fibroblasts

Fibroblasts are cells with spindle-formed morphology which are easy to identify but are quite indistinctly defined on a molecular basis. This makes it difficult to define these cells in comparison to macrophages or endothelial cells for example. They are present in every organ differing significantly in a site-specific manner [46]. Tumor- or cancer-associated fibroblasts (CAFs) show a highly activated state that is induced by growth factors with transforming growth factor (TGF) as the most prominent one and expresses the highly contractable α smooth muscle actin (αSMA; also known as ACTA2) representing a so-called “myofibroblastic” phenotype [46,47]. Additionally, this heterogenous intra-tumoral subpopulation can promote angiogenesis by the production of VEGF [48] and are involved in metabolic reprogramming, as well as resistance towards hypoxic stress in tumor tissues [49].

Since there is still a lack of specific CAF markers during the clinical routine, the histopathological differentiation is done by their typical spindle-like shape and the absence of “classical” endothelial, epithelial, or leukocyte markers. Interestingly, the exact origin of CAFs is also an ongoing debate. Most of CAFs are supposed to differentiate from normal local fibroblasts at the tumor site during a process called “stromagenesis” resembling that the cellular dysfunction not only affects the tumor cells but also the stroma in which these cells are embedded [47,50]. However, CAFs may also develop from bone marrow-derived mesenchymal stem cells (MSCs) since experimental in vivo data suggest the ability of MSCs to change into CAFs [51]. Astonishingly, even adipocytes might be able to convert into CAFs as this phenomenon has been described before [52]. Other cell types from which CAFs may derive are pericytes or endothelial cells [47]. Epithelial-to-mesenchymal (EMT) transition from tumor cells into the activated CAFs has been discussed, too.

So far, only little is known about the molecular function of CAFs within both EAC and ESCC. However, there is more and more evidence that these cells may play a crucial role within the patients’ prognosis reflecting unfavorable tumor biology. In 2013, Schoppmann and colleagues first described the negative prognostic relevance of CAFs within a cohort of 200 EAC patients being associated with worse tumor stage, as well as a higher rate of nodal metastasis [53]. Especially those CAFs with myofibroblastic phenotype seem to predict a poor outcome as patients with αSMA-positive CAFs show impaired postsurgical survival [54]. Interestingly, Hanley and coworkers could demonstrate that the underlying fibroblast-to-myofibroblast transdifferentiation was depending on intracellular reactive oxygen species generated by NOX4 and that pharmacological blockage of this enzyme caused decreased αSMA, inhibited positive myofibroblastic CAF formation, and slower tumor growth in both in vitro and in vivo models [55]. Thus, this targeted pharmacological stroma manipulation might reveal novel therapeutic options in cancer treatment.

Different CAF populations within EC seem to underlie selection processes, such as therapeutic pressure. In a mixed study cohort of both EC entities (including EAC and ESCC), SPARC-positive CAFs were enriched after neoadjuvant chemotherapy compared to decreased numbers of COL11A1-positive CAFs [56]. In this way, certain subclones might serve as putative targets for upcoming therapeutic approaches or as makers for treatment prediction in EC of both histopathological subtypes. Additionally, patients with a high intra-tumoral stroma ratio show lower to no response towards neoadjuvant chemoradiation compared to patients with only low stroma expression [57]. On the other hand, neoadjuvant therapy itself may induce CAF-depending resistance since it has been reported that chemoradiation in EAC patients might stimulate increasing autocrine TGF-β production within the epithelial tumor cells resulting in a higher rate of EMT [58].

Recently, it has been reported that inhibition of Vimentin and Nf-κB as relevant mediators for carcinogenesis within the myofibroblasts of a Barrett’s esophagus mouse model prohibits the progression into dysplastic epithelium [59]. Blocking the interleukin-6 (IL-6) crosstalk between CAFs and epithelial tumor cells negatively affects tumor growth in vitro as IL-6 might mediate the EMT in both subtypes, EAC and ESCC [60,61] via autocrine and paracrine secretion of this cytokine. Several molecular pathways have been suggested to be involved in EMT including the PTEN/Akt and MEK/Erk or FOXO1/TGFβ1signaling and CXCL1 secretion [62,63,64] as putative mechanisms for CAF-associated chemoresistance especially in ESCC. In addition, microRNAs (miRs) such as miR-27 seem to be able activating CAFs in a TGF-β depending manner [65].

Another relevant feature of CAFs is their ability to remodel the extracellular matrix stiffness within the tumor microenvironment [66]. Interacting with matrix metalloproteinases (MMPs) (e.g., MMP-2, -3, -7, and -9) and influencing the collagen fiber content within the tumor tissue, this subgroup of cells directly affects such crucial aspects as tumor formation, progression, or metastasis [67]. Recently, it has been shown that metastasis-associated fibroblasts (MAFs) lead to stiffening of the extracellular matrix within hepatic metastases of colorectal cancer, causing increased angiogenesis and anti-angiogenic therapy resistance [18]. Interestingly, renin-angiotensin (RAAS) inhibition reduced MAF activity and, therefore, impaired the stiffness within the metastasis supporting the results of anti-angiogenic therapy in vivo [18]. However, it is unclear if those results can be transferred to esophageal cancer since ambiguous data have been published. On the one hand, RAS factors, such as angiotensin-converting enzyme (ACE) and the angiotensin II subtype 1 receptor (AT1R), seem to be upregulated in Barrett’s esophagus [68] but, on the other hand, no prognostic effects of RAS-inhibition have been found in both ESCC and EAC so far [69].

**Figure 2:**
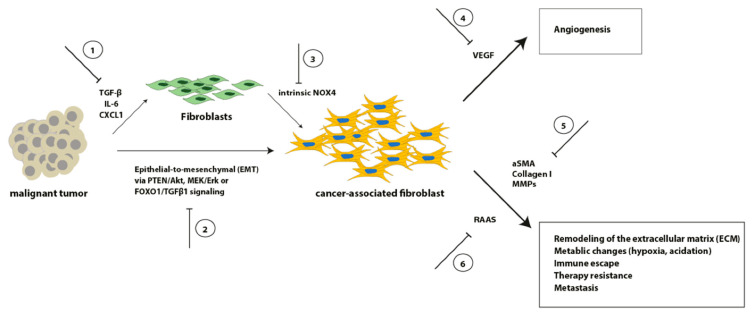
Schematic overview of selected signaling pathways involved in the transformation from fibroblasts into CAFs, as well as representative CAF effects on crucial tumor aspects. Inhibition of these processes (**1**–**6**) might result in novel therapeutic options.

Although we do not fully understand all interactions of CAFs with other cellular subpopulations within the intra-tumoral environment, it becomes more and more obvious that stroma-specific manipulation might be a novel therapeutic approach for treatment of ESCC and EAC in the future (Figure 2).

## 4. Tumor-associated Macrophages

Infiltration of tumor-associated immune cells is one of the hallmarks of cancer [70]. A significant amount of these cells is represented by macrophages; accordingly, nearly every solid tumor is heavily infiltrated by macrophages [71,72,73,74]. To understand the role of macrophages in tumors it is helpful to look at how macrophages function in wound healing. In the early phase of a wound, macrophages clear the wound of debris and bacteria and recruit other immune cells to help repair the damaged tissue. These tasks are summarized under the term ‘pro-inflammatory’. Next, macrophages help to rebuild the tissue by fostering angiogenesis and re-epithelization by producing granulation tissue. In the last phase of wound healing, they help to limit immune responses of other cells, remodel the tissue, and clear apoptotic cells. For these different processes, macrophages need different states of activation termed polarization [75]. These different polarization states are simplified in M1 or M2 macrophages. This nomenclature is based on inflammation and immunity also used for tumor-associated macrophages often with the addition ‘M1/M2-like’. M1-macrophages are believed to be more in a pro-inflammatory state, while M2-like macrophages are immune-suppressive, at the same time pro-angiogenic, and express metalloproteases to degrade basement membranes and other extracellular matrix structures to foster invasion and migration [76,77]. Polarization states can be determined by distinct chemokine expression and immune receptor expression patterns [78,79] triggered by a chaotic milieu of an exponentially expanding malignant lesion. The functional and structural abnormal vascular system that lacks a hierarchic architecture does not provide proper delivery of oxygen and nutrients and shows insufficient clearance of metabolic waste and carbonic dioxide which leads to a hostile environment where normoxic, hypoxic, and necrotic tissue is in the constant remodeling process. Dying cells secrete chemokines which lead together with that harsh environment to the recruitment of macrophages, that similar to the early phase of a wound start to (try to) repair ‘the wound that never heals’ [80].

Accumulation of tumor-associated macrophages in human solid tumors is correlated in most but not every entity with a poor prognosis [55,56]. In some entities, e.g., colorectal cancer the implication of intra-tumoural immune cells seems to change throughout disease progression [31,35,81,82]. Tumor macrophages in most advanced cancers execute key functions of tumor progression by secreting growth factors that directly stimulate tumor cell proliferation and survival, by fostering angiogenesis by secreting pro-angiogenic cytokines and producing extracellular components of angiogenesis like, e.g., collagen IV, by promoting tumor cell invasion via degradation of basement membranes and other extracellular matrix by metalloproteinases and by mediating adaptive immunity via immunomodulatory or immunosuppressive stimuli [70,73].

Li et al. published a meta-analysis concerning the impact of tumor-associated macrophages in esophageal cancer. The studies included in this analysis were exclusively from Asia and reported on ESCC, besides one study from the US [83]. The meta-analysis found infiltration with M2-macrophages as significantly relevant for overall survival. Infiltration with M2-macrophages contributed to poor survival and increased TNM stage in ESCC. Interestingly, high infiltration of M2-macrophages to ESCC is also associated with poor prognosis after and poor pathological response to neoadjuvant treatment [84].

EAC is a disease that is at least initially driven by chronic inflammation, due to reflux disease with metaplasia of the distal esophagus with a significant upregulation of inflammatory cytokines which can influence the prognosis [85]. It is not surprising that immune cells are deeply involved in promoting malignant progression [86]. In EAC, M2-macrophage infiltration, specifically, a high M2/M1-like ratio was accompanied by poor prognosis [87]. Interestingly, this was only relevant in treatment naïve patients and the observed role of macrophages was not detectable after neoadjuvant treatment.

Nevertheless, both in EAC and ESCC tumor-infiltrating macrophages seem to play a pivotal role in malignant progression and therapy resistance [88] and represent a potentially valuable therapeutic target to further increase pathological response and overall survival. Though, targeting macrophages has not entered clinical practice as rapidly as other targeted concepts due to the complexity and diversity of TAM function and phenotype. Table 1 gives an overview of recent Phase I trials evaluating macrophage targeted therapies.

## 5. T-Cells and Myeloid-Derived Suppressor Cells, Immunotherapy

In addition to monocytes, mast cells, myeloid progenitors, and macrophages, T-cells compose a significant part of the tumor immune cell infiltrate in most solid tumors. CD-8^+^ cytotoxic T-lymphocytes, CD4^+^ T_h_1 helper T cells, and natural killer cells are critical players in eliminating malignant cells in the healthy human organism which has widely been demonstrated in mice models where mice lacking these cells or subsets of them have a significantly higher susceptibility to develop malignancies [70,92]. Tumors develop effective strategies to avoid such elimination by the immune system. To regain effective T cell-mediated anti-tumor activity became one of the most applied targeted therapy concepts of modern cancer treatment namely checkpoint inhibition. Programmed cell death protein 1 (PD-1) is a so-called immune checkpoint protein expressed on the cell surfaces of lymphocytes. Tumor cells express programmed cell death ligand 1 (PD-L1) which mediates prevention of cytotoxic anti-tumor t cell activity by inducing apoptosis in antigen-specific T cells and by interfering with regulatory T cells [93,94]. Drugs that prevent PD-1/PD-L1 interaction, e.g., pembrolizumab, a humanized monoclonal anti-PD-1 antibody, were developed with highly promising results in several tumor entities.

In ESCC, PD-L1 expression by tumor cells is an independent prognostic factor predicting worse outcomes in PD-L1 positive patients [95]. In line with this, the Keynote-181 study, a randomized phase III trial involving over 600 patients with esophageal cancer including a mixed cohort of both, ESCC and EAC patients showed that pembrolizumab lead to a significant survival benefit compared to chemotherapy (investigator’s choice of paclitaxel, docetaxel, or irinotecan) in patients with ESCC. Interestingly, patients with EAC showed no survival benefit [96]. These results were in line with both, the Keynote-061 trial, where Pembrolizumab failed to show any effect in gastro-esophageal junction adenocarcinoma [97], and the Attraction-3 study, where Nivolumab, another anti-PD-1 antibody, significantly prolonged overall survival compared to chemotherapy in ESCC patients [98]. These large clinical trials show that in ESCC immune checkpoint blockade is a valuable treatment approach that is as a single-agent even superior to chemotherapy in a palliative setting, but in EAC these drugs still have to show efficacy. Whether and how these differences are determined by the cancer cell itself or complex microenvironmental cues that manipulate the immune response beyond PD-L1/PD-1 signaling has to be further elucidated. That the latter is a likely scenario is supported by findings that high amounts of intratumoral CD8^+^ T cells have been shown to be associated with prolonged survival in both ESCC and EAC [99]. Another study demonstrated that high abundance of CD8^+^ T cells was accompanied by high PD-L1 expression and that both factors were beneficial for patients survival in esophagogastric junction and gastric adenocarcinomas [100]. A factor independent of PD-L1/PD-1 expression status might be the abundance and activity of myeloid-derived suppressor cells (MDSCs) that are known to critically interfere with adaptive anti-cancer immune responses in several ways [101,102,103]. High infiltration counts of MDSCs are associated with detrimental outcome parameters in esophageal cancer patients’ and MDSCs promote esophageal cancer growth in experimental disease models [104,105]. A recent work has done great efforts to characterize the immune-suppressive landscape in esophageal cancer at single-cell resolution by transcriptome analysis of tumor-infiltrating immune cells [106]. Unfortunately, this work was limited to ESCC, similar data are urgently needed for EAC patients. This would potentially clarify the differential response to immunotherapy between EAC and ESCC patients. This is highly clinically relevant to improve and individualize this therapeutic concept in the future. Furthermore, results from trials that incorporate immunotherapy into neoadjuvant regimens in esophageal or esophagogastric junction cancer (NCT04159974, NCT03421288) are eagerly awaited to clarify safety and efficacy and whether immunotherapy can improve response to neoadjuvant treatment, which is highly relevant for overall survival, particularly in EAC.

## 6. Conclusions

During recent decades a considerable amount of knowledge concerning different cellular and extracellular compartments within the tumor microenvironment of esophageal cancer has been gained. Instead of merely focusing on the epithelial tumor cells, it becomes more and more obvious to consider other intra-tumoral cell populations, as well as multiple interactions between these populations.

Based on this knowledge targeted therapies have been developed that mostly in conjunction with conventional chemotherapy aim to advance treatment efficacy. Although significant improvement has been reached, treatment responses and overall survival in esophageal cancer patients is still poor. Major challenges remain in further improving established therapeutic concepts and (re-)evaluating them in certain clinical situations as, e.g., ramucirumab in the neoadjuvant setting.

Another very interesting question is the differences in sensitivity of EAC and ESCC towards immunotherapy. To uncover potential causes might elicit new modes of resistance and chances for adjustments to checkpoint inhibition in esophageal cancer.

Finding suitable biomarkers of response and resistance is a highly relevant challenge. This is true for every oncologic treatment concept, but particular for targeted therapies due to the high cost for health care systems and society. Very few markers exist or proceeded to daily clinical practice in solid tumors which highlights the urgent need for further research here.

The vaguest but probably also the most exciting challenge is to explore novel concepts based on molecular findings regarding the regulation of the tumor microenvironment. Thorough basic and translational research that also reports potential risks of potential novel targeted therapies is required.

## Figures and Tables

**Table 1:** Overview of macrophage targeting therapies in clinical Phase I trials. Please note that the last column indicates inclusion or eligibility of esophagogastric cancers or ESCC (+) or not (−).

Drug	Targeted Mechanism	Stage Towards Clinical Application, Reference	Including EAC/ESCC
Carlumab	CCL2 Inhibition	Phase I [89]	+
Vanucizumab	VEGF/ANG-2 Inhibition	Phase I, NCT02665416	−
CP-870,893	CD40 Agonism	Phase I [90]	−
AMG820	CSF-1R Inhibition	Phase I [91]	−
LY3022855	CSF-1R Inhibition	Phase I NCT02718911	+
EF-022 (Efranat)	Modified vitamin-D-binding protein (macrophage-activating factor)	Phase I NCT02052492	+
PLX7486	CSF-1R Inhibition	Phase I NCT01804530	+
